# Supplementary material for: Omics-based construction of regulatory variants can be applied to help decipher pig liver-related traits
Source: Commun Biol. 2024 Mar 29;7:381. doi: 10.1038/s42003-024-06050-7 (PMC10980749; doi:10.1038/s42003-024-06050-7)
Supplement: Supplementary file 2 — Supplementary Information [file 42003_2024_6050_MOESM2_ESM.pdf]

Supplementary Figures 1-9

Supplementary Fig. 1: Quality control of samples and features of H3K27ac peaks.

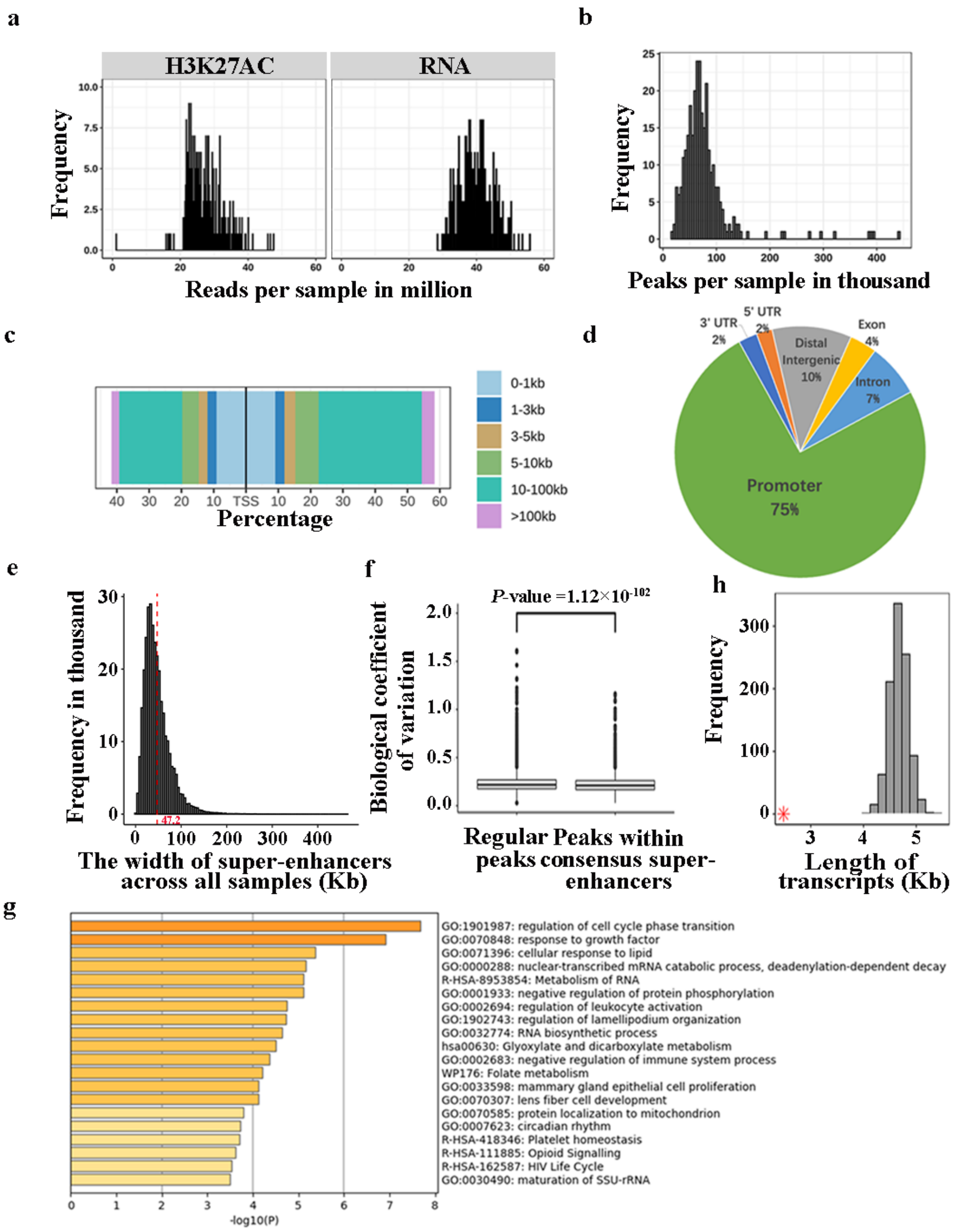

a The number of uniquely mapped reads for each sample in ChIP-seq and RNA-seq experiments. The x-axis indicates the read count, with 292 samples for ChIP-seq and 256 samples for RNA-seq. **b** The frequency distribution of the number of peaks identified in each ChIP-seq sample. **c** The distribution of consensus peaks relative to the TSS of the nearest gene. The x-axis represents the percentage of peaks. **d** The genomic positions of the 5,000 most active consensus peaks and promoter regions exhibit dominant proportion. **e** The distribution of the width of super-enhancers identified in each ChIP-seq sample. The mean width is 47.2 kb, as indicated by the red dashed line. **f** The comparison of the variation of peaks across samples between those

within consensus super-enhancers and regular peaks located outside of these regions. The statistical significance of this comparison was determined using the t-test, and the resulting *P*-values are presented. The boxplots display the median, the 25th and 75th percentiles. The whiskers indicate the minimum and maximum values, and outliers are shown as points outside the ends of the whiskers. **g** The enrichment pathway analysis on a set of 237 genes that are covered by 43 consensus super-enhancers with activity in at least 99% of samples. Metascape was used to conduct the enrichment analysis. **h** Similar to Fig1 F, but for transcript length. The average length of polyadenylated eRNA transcripts is indicated with a red asterisk. The identified polyadenylated eRNAs are shorter than annotated genes, and significance was determined using 1000 permutations (*P*-value = 9.99×10^{-4}).

Supplementary Fig. 2: Estimates of heritability of H3K27ac peaks.

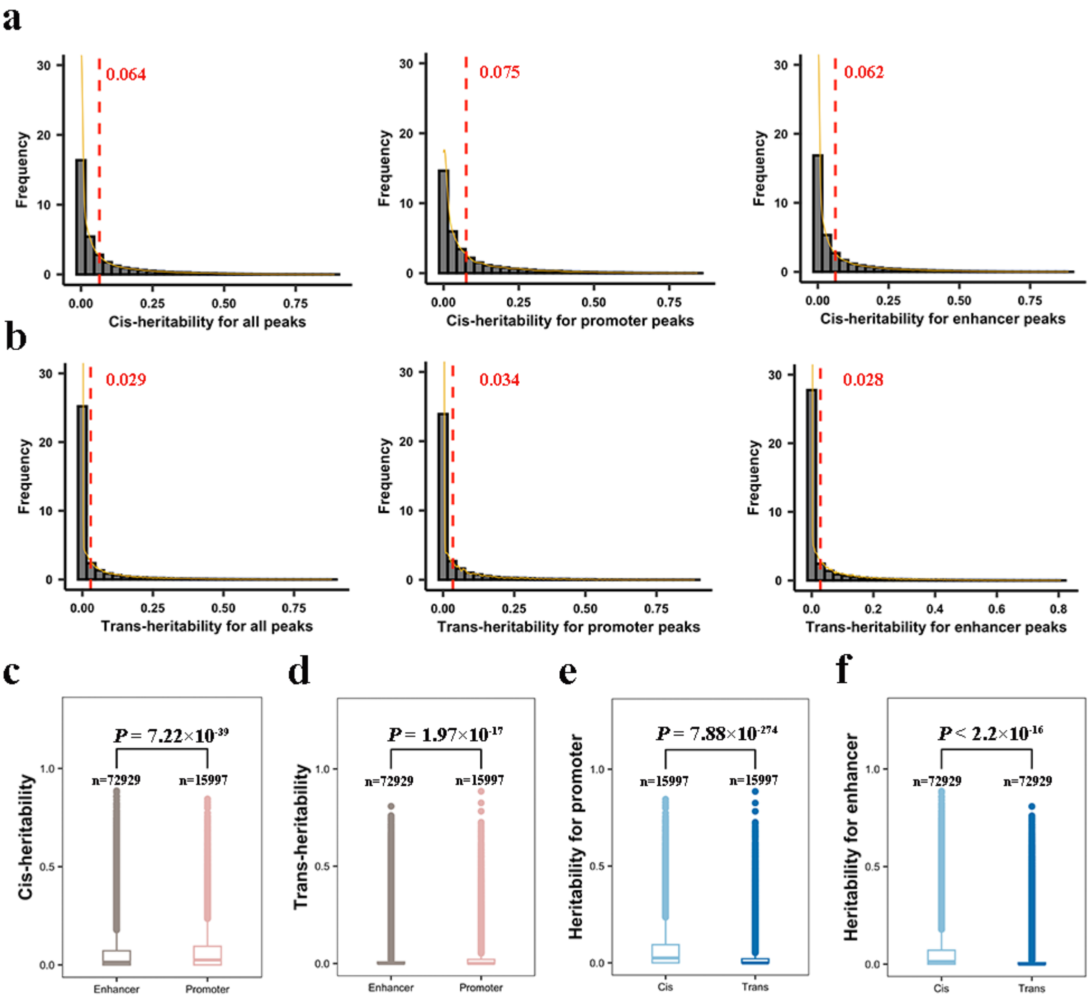

a Distribution of cis-heritability for all peaks, promoter peaks, and enhancer peaks. The average estimates of cis-heritability were shown with the red dashed line. **b** Distribution of trans-heritability for all peaks, promoter peaks and enhancer peaks. The average estimates of trans-heritability were shown with the red dashed line. **c** Comparison of cis-heritability for enhancer and promoter peaks. **d** Comparison of trans-heritability for enhancer and promoter peaks. **e** Comparison of cis-heritability and trans-heritability for promoter peaks. **f** Comparison of cis-heritability and trans-heritability for enhancer peaks. The boxplots display the median, the 25th and 75th percentiles. The whiskers indicate the minimum and maximum values, and outliers are shown as points outside the ends of the whiskers.

Supplementary Fig. 3: Data processing workflow and properties of acQTLs.

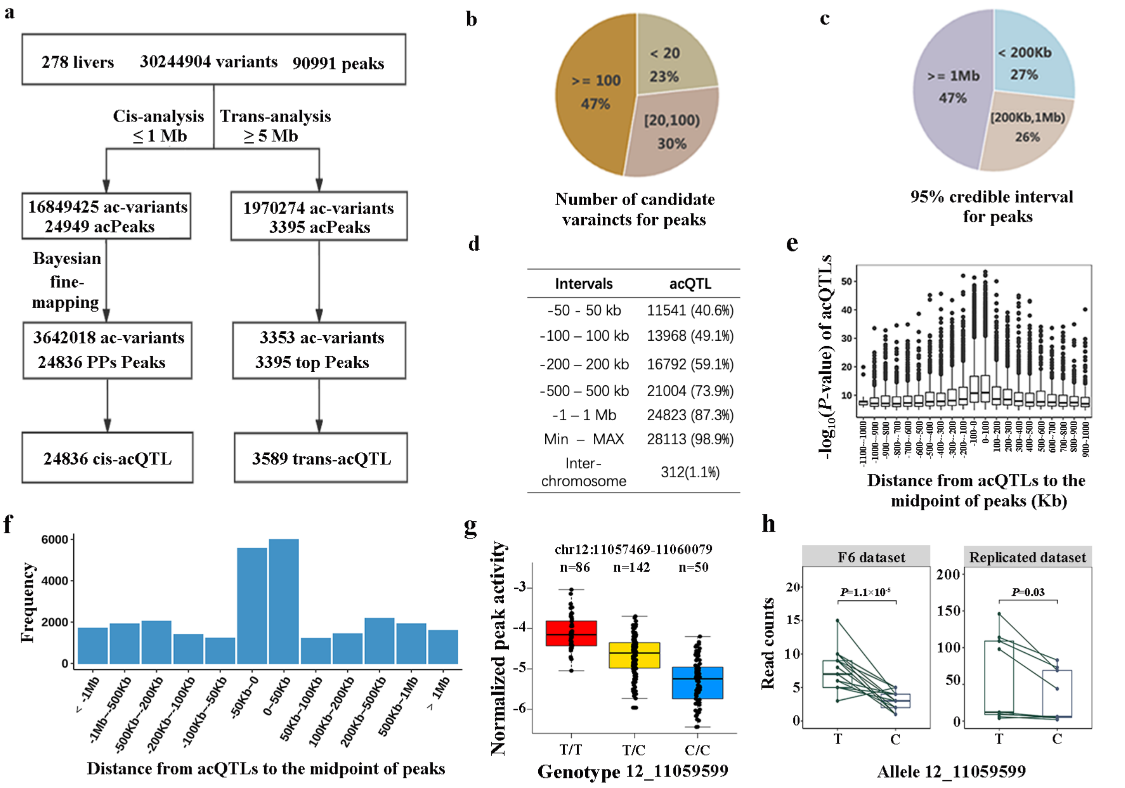

a Schematic workflow outlines the acQTL mapping process, which involves both cis- and trans-analysis. In the cis-analysis, genomic variants within 1 Mb of the first base of the consensus peaks are selected. In contrast, the trans-analysis focuses on genomic variants more than 5 Mb away from the same starting point. **b** The number of candidate variants of consensus peaks in the cis-acQTL fine-mapping analysis. **c** 95% credible interval for candidate variants of consensus peaks in the cis-acQTL fine-mapping analysis. **d** The proportion of acQTLs within each distance interval between the acQTLs and the midpoint of the target peaks. **e** The -log$_{10}$(*P*-value) distribution of acQTLs within each distance interval between the acQTLs and the midpoint of the target peaks. **f** The frequency distribution of acQTLs within each distance interval between the acQTLs and the midpoint of the target peaks. Distance intervals are classified based on upstream or downstream. **g-h** Another replicated lead acQTL variant in allelic imbalances analysis and related to Fig 2A and 2B. The lead acQTL variant 12_11059599 reduce the peak activity of chr12:11057469-11060079 when changing from T to C. The boxplots display the median, the 25th and 75th percentiles. The whiskers indicate the minimum and maximum values, and outliers are shown as points outside the ends of the whiskers. **g** Genotype-dependent variations are observed in peak activity. The x-axis represents genotypes, and the y-axis shows the normalized peak activity. The number of samples is denoted above each box. **h** Imbalanced read coverage of replicated alleles in the F6 dataset and the replicated dataset for allele 12_11059599. Allele T has a higher read coverage than C in both datasets, and the t-test *P*-value is denoted.

Supplementary Fig. 4: Data processing procedure, eQTL characteristics, and a comparison of acQTLs and eQTLs.

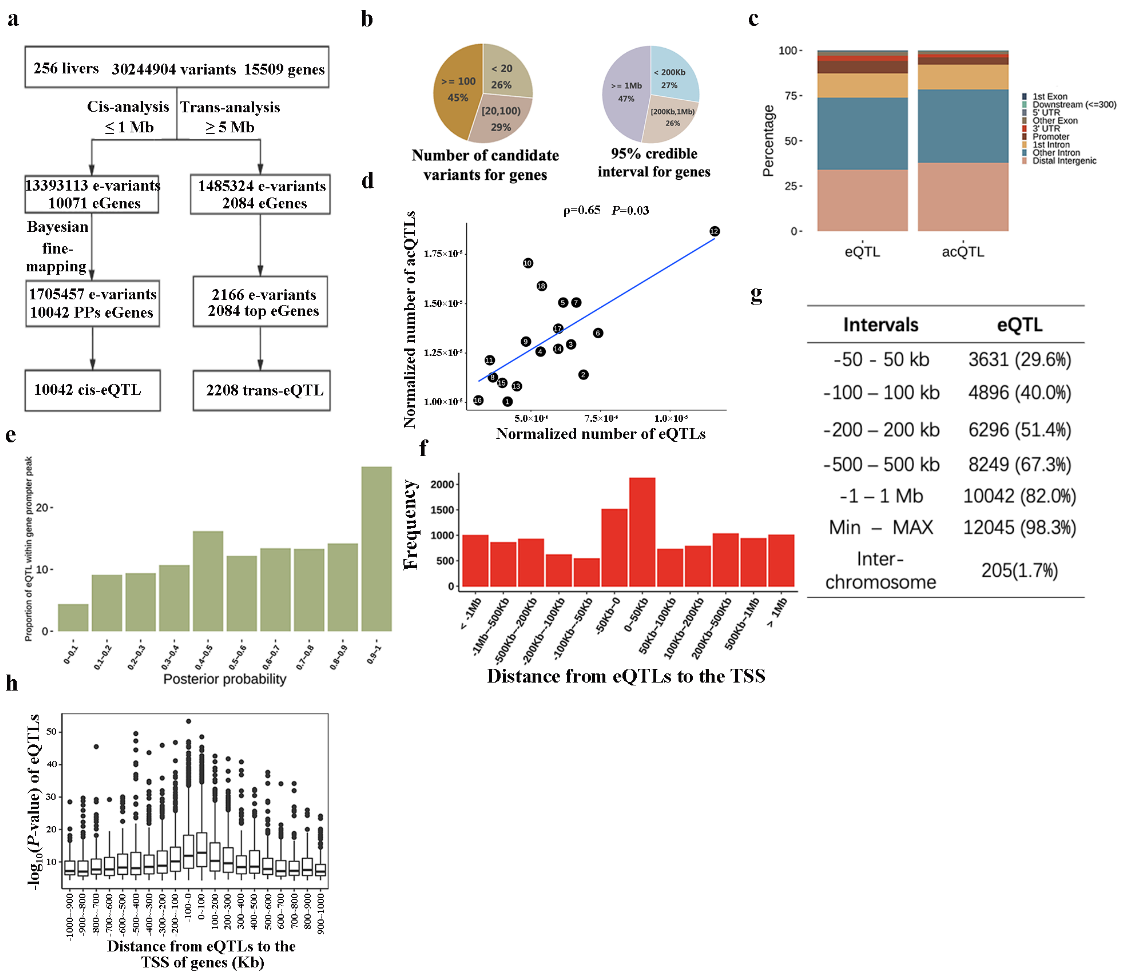

a A workflow diagram illustrates the eQTL mapping procedure, which includes both cis- and trans-analysis. In the cis-analysis, genomic variants within 1 Mb of the TSS of genes are chosen. The trans-analysis, on the other hand, concentrates on genomic variants that are more than 5 Mb away from the TSS. **b** The number and 95% credible interval of candidate variants of genes in the cis-eQTL fine-mapping analysis. **c** The genomic distribution comparison between eQTLs and acQTLs. EQTLs and acQTLs exhibit a comparable proportion. **d** The correlation between the normalized number (adjusting for chromosome length) of eQTLs and acQTLs across different chromosomes, with each chromosome represented by a black dot. The *P*-value is denoted (Spearman test, ρ=0.65, *P*-value=0.03). **e** The proportion of cis-eQTLs located in promoter H3K27ac peaks of target genes within each Bayesian posterior probabilities interval. The x-axis represents intervals, and the y-axis represents proportion. **f** The frequency distribution of eQTLs within each distance interval between the eQTLs and the TSS of genes. Distance intervals are classified based on upstream or downstream. **g** The proportion of eQTLs within each distance interval between the eQTLs and the TSS of genes. **h** The $-\log_{10}(P\text{-value})$ distribution of eQTLs within each distance interval between the eQTLs and the TSS of genes. The boxplots display the median, the 25th

and 75th percentiles. The whiskers indicate the minimum and maximum values, and outliers are shown as points outside the ends of the whiskers.

Supplementary Fig. 5: Comparison of the chromosomal distribution of eQTLs and acQTLs.

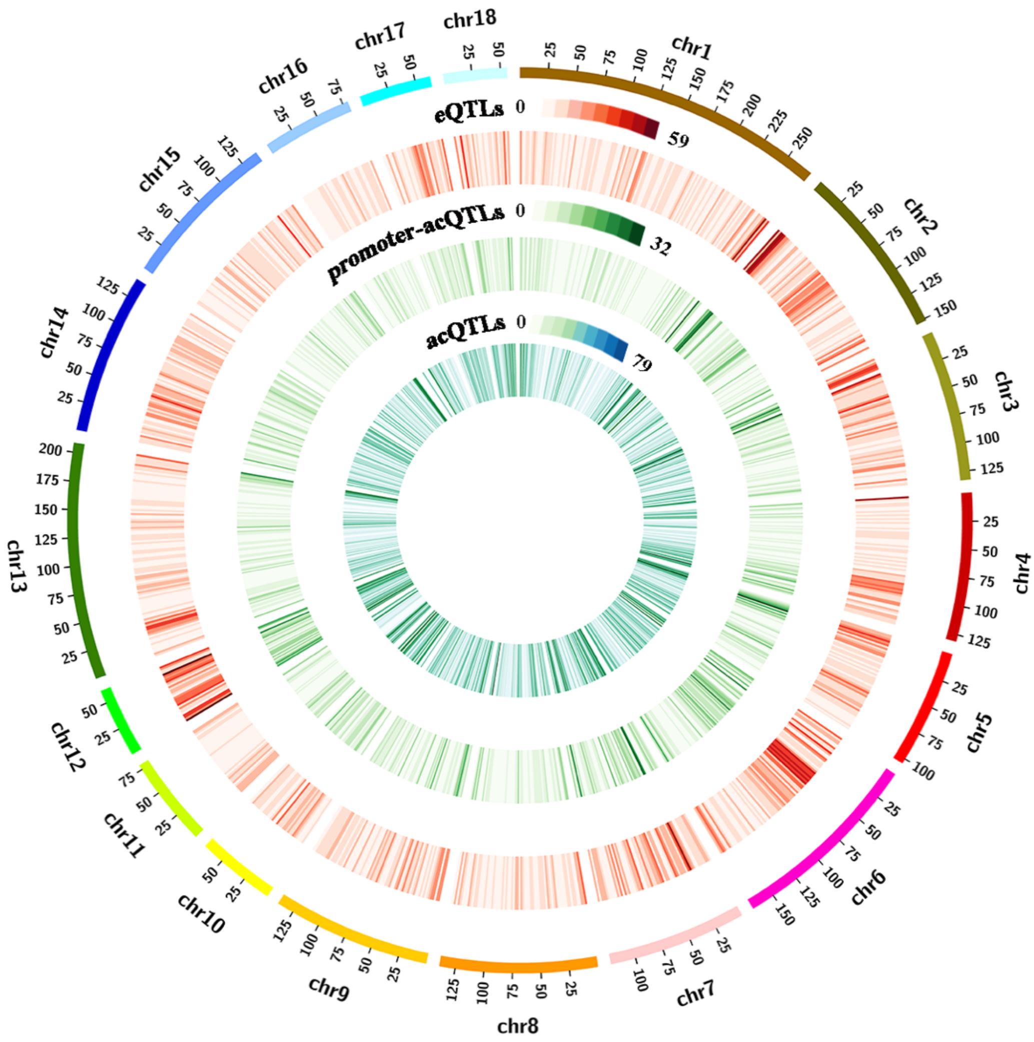

QTLs whole-genome CIRCOS profiles of RNA and H3K27ac. The circles represent the distribution of chromosomes, eQTLs, promoter-acQTLs, and acQTLs, from outer to inner. Promoter-acQTLs are the acQTLs associated with promoter peaks. Each chromosome is labelled in units of 1 Mb. The number of QTLs is calculated by dividing each chromosome into 2 Mb windows. The distribution of promoter-acQTLs will be similar to the distribution of eQTLs a bit more than that of acQTLs.

Supplementary Fig. 6: Colocalization of acQTLs and eQTLs.

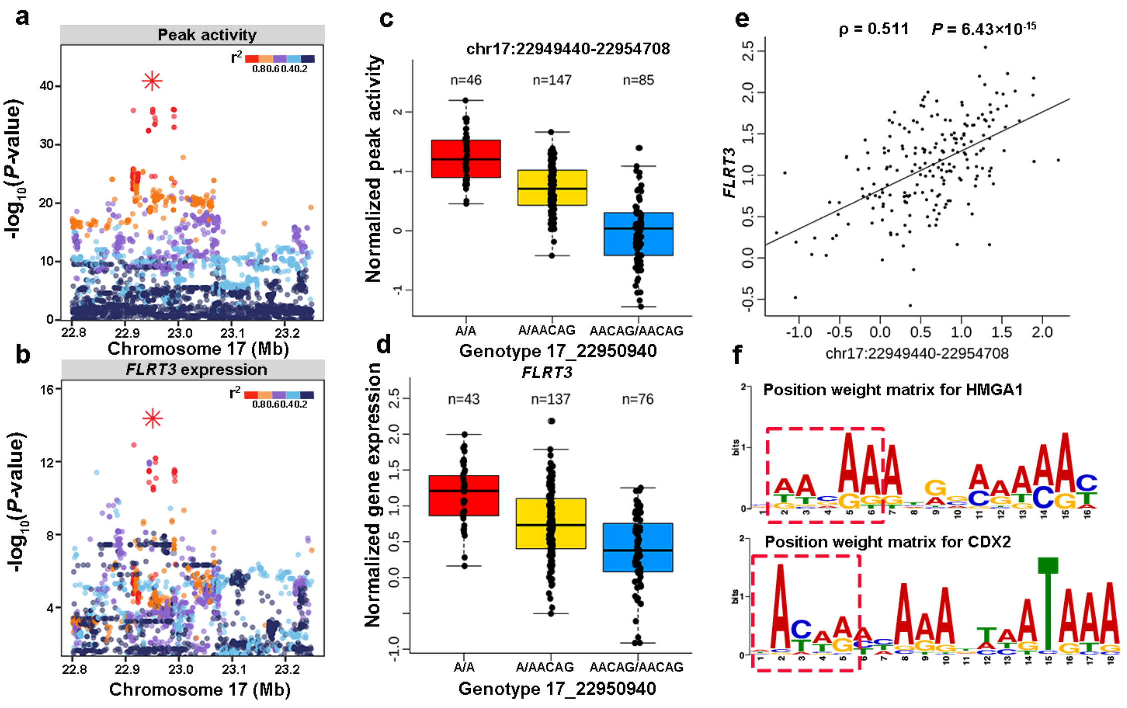

a-f An example of QTL 17_22950940 that simultaneously influences the peak activity of chr17:22949440-22954708 and the gene expression of *FLRT3*. **a** AcQTL association plot for the peak chr17:22949440-22954708. The x-axis represents genome position, and the y-axis represents the -$\log_{10}$(*P*-value) of the significance of the association. Each dot represents a variant of the genome. The acQTL 17_22950940 is denoted by an asterisk and the pairwise r^2 between the acQTL and all genomic variants is indicated by distinct colors. **b** EQTL association plot for the gene *FLRT3*. The x-axis represents the genomic range equivalent to **a**. The eQTL 17_22950940 is denoted by an asterisk and the pairwise r^2 between the eQTL and all genomic variants is indicated by distinct colors. **c** The effect of the genotype 17_22950940 on the peak activity. The x-axis represents three genotypes of 17_22950940, and the y-axis shows the normalized peak activity of chr17:22949440-22954708. The number of samples is denoted above each box. **d** The effect of the genotype 17_22950940 on the gene expression. The x-axis represents three genotypes of 17_22950940, and the y-axis shows the normalized gene expression of *FLRT3*. The number of samples is denoted above each box. The boxplots display the median, the 25th and 75th percentiles. The whiskers indicate the minimum and maximum values, and outliers are shown as points outside the ends of the whiskers. **e** The correlation plot between the gene expression of *FLRT3* and the peak activity of chr17:22949440-22954708. The x-axis and the y-axis represent the normalized signals for the peak and the gene, respectively. The solid black line represents the fitted curve, and the corresponding correlation coefficient and *P*-value are denoted (Spearman test). **f** Position weight matrix (PWM) for HMGA1 and CDX2, with the acQTL/eQTL 17_22950940 indicated by red dashed boxes.

Supplementary Fig. 7: Identification of sub-threshold GWAS loci, regulatory elements and target genes for phosphatidylcholine (PC) (16:0/16:0).

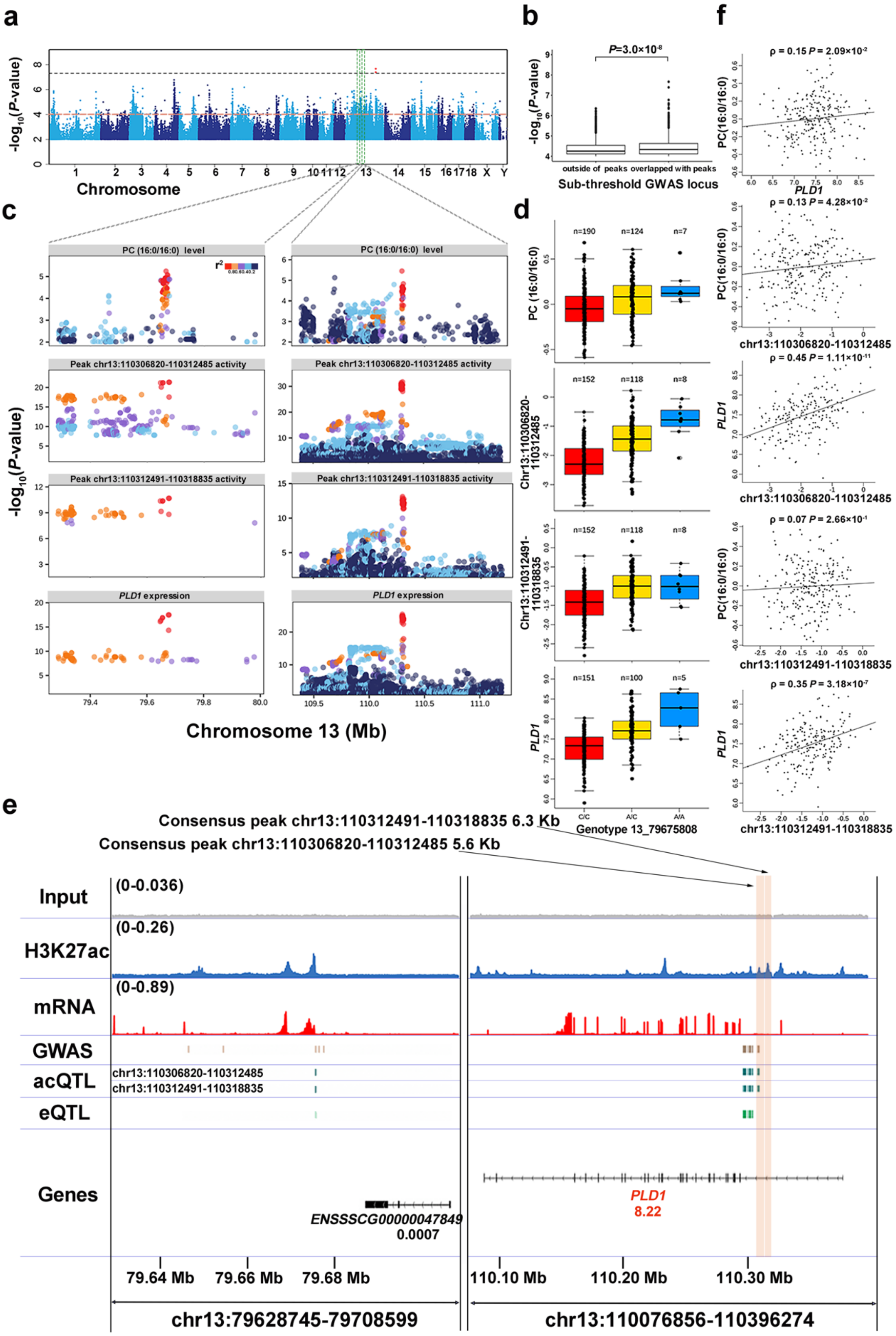

a-f Similar to Fig. 4, the panels show the results for the trait PC (16:0/16:0). The significance threshold for GWAS was changed to 1×10^{-4} in order to search for sub-

threshold functional loci that are frequently neglected due to modest effect sizes. Two independent sub-threshold GWAS loci associated with the same two peaks (chr13:110306820-110312485 and chr13:110312491-110318835) and the same gene (*PLD1*). **a** Manhattan plot (−log10(*P*-value) genome-wide association plot) of a genome-wide association study (GWAS) on PC (16:0/16:0). The x-axis represents the different chromosomes, and the y-axis represents the -log10(*P*-value) of variants. The empirical genome-wide significance threshold 5×10^{-8} is represented by a dark dashed line. All variants that exceed the empirical genome-wide significance threshold are colored red. The adjust genome-wide significance threshold 1×10^{-4} is represented by an orange dashed line. The two independent sub-threshold GWAS loci are highlighted with green dashed boxes. **b** Variants within sub-threshold GWAS loci that overlap H3K27ac peaks have significantly greater association signals than variants within loci outside H3K27ac peaks. The x-axis represents the type of sub-threshold GWAS loci and the y-axis represents the -log10(*P*-value) of variants. *P*-value is denoted above boxes (T-test). **c** Association plots for four phenotypes (phosphatidylcholine PC (16:0/16:0), peak chr13:110306820-110312485, peak chr13:110312491-110318835 and gene *PLD1*) within two independent sub-threshold GWAS loci. The x-axis represents the genomic range, and the y-axis represents the -$\log_{10}$(*P*-value) for the association analysis. One part shows the sub-threshold GWAS loci that contain the SNP 13_79675808, that operates as both a trans-acQTL (for peak chr13:110312491-110318835 and peak chr13:110306820-110312485) and a trans-eQTL (for gene *PLD1*). Another part shows the sub-threshold GWAS loci that contain the cis-acQTL 13_110297901 for peak chr13:110312491-110318835, the cis-acQTL 13_110310074 for peak chr13:110306820-110312485 and cis-eQTL 13_110299400 for gene *PLD1*. The pairwise linkage disequilibrium (r^2) between the lead variant for the corresponding molecular phenotype and all genomic variants within that region is indicated by distinct colors. **d** The lead SNP 13_79675808 with the highest significance functions as both a trans-acQTL and a trans-eQTL. The x-axis shows the three genotypes and the y-axis represent the normalized signals for three molecular phenotypes. The number of samples is denoted above each box. The boxplots display the median, the 25th and 75th percentiles. The whiskers indicate the minimum and maximum values, and outliers are shown as points outside the ends of the whiskers. **e** Genome browser views of the comprehensive molecular profile within two independent sub-threshold GWAS loci. The x-axis displays the genomic range, and the y-axis contains tracks of data. One part shows the genomic range chr13:79628745-79708599 containing SNP 13_79675808, that operates as both a trans-acQTL and a trans-eQTL. Another part shows the genomic range chr13:110076856-110396274 containing the cis-acQTLs (13_110297901 and 13_110310074) and the cis-eQTL (13_110299400). The normalized read depths for input, H3K27ac, and mRNA are denoted in brackets. The GWAS track displays variants exceeding *P*-value 1×10^{-4} from GWAS analysis and showing high LD (r^2 > 0.8) with acQTLs. The acQTL and eQTL track from the panel shows SNP 13_79675808 with highest significance for the consensus peaks (chr13:110306820-110312485 and chr13:110312491-110318835) and the gene *PLD1*. The acQTL and eQTL track from the panel shows candidate variants within 95% confidence interval for the consensus peaks

(chr13:110306820-110312485 and chr13:110312491-110318835) and the gene *PLD1* based on fine-mapping analysis. The consensus peaks are highlighted by transparent orange rectangles. The gene track depicts the positions and orientations of annotated genes, with exons represented by black boxes. The TPM values for each gene are displayed below their respective names, with *PLD1* gene highlighted in red. The genomic annotations utilized were sourced from the Ensembl database (version 1.98 of the pig GTF file). **f** The pairwise correlation plot of molecular phenotypes. The x-axis and the y-axis represent the normalized signals for each molecular phenotype. The solid black line represents the fitted curve, and the corresponding correlation coefficient and *P*-value are denoted (Spearman's correlation).

Supplementary Fig. 8: Prioritizing functional variants, genes and regulatory elements for published GWAS of hematocrit and red blood cell count.

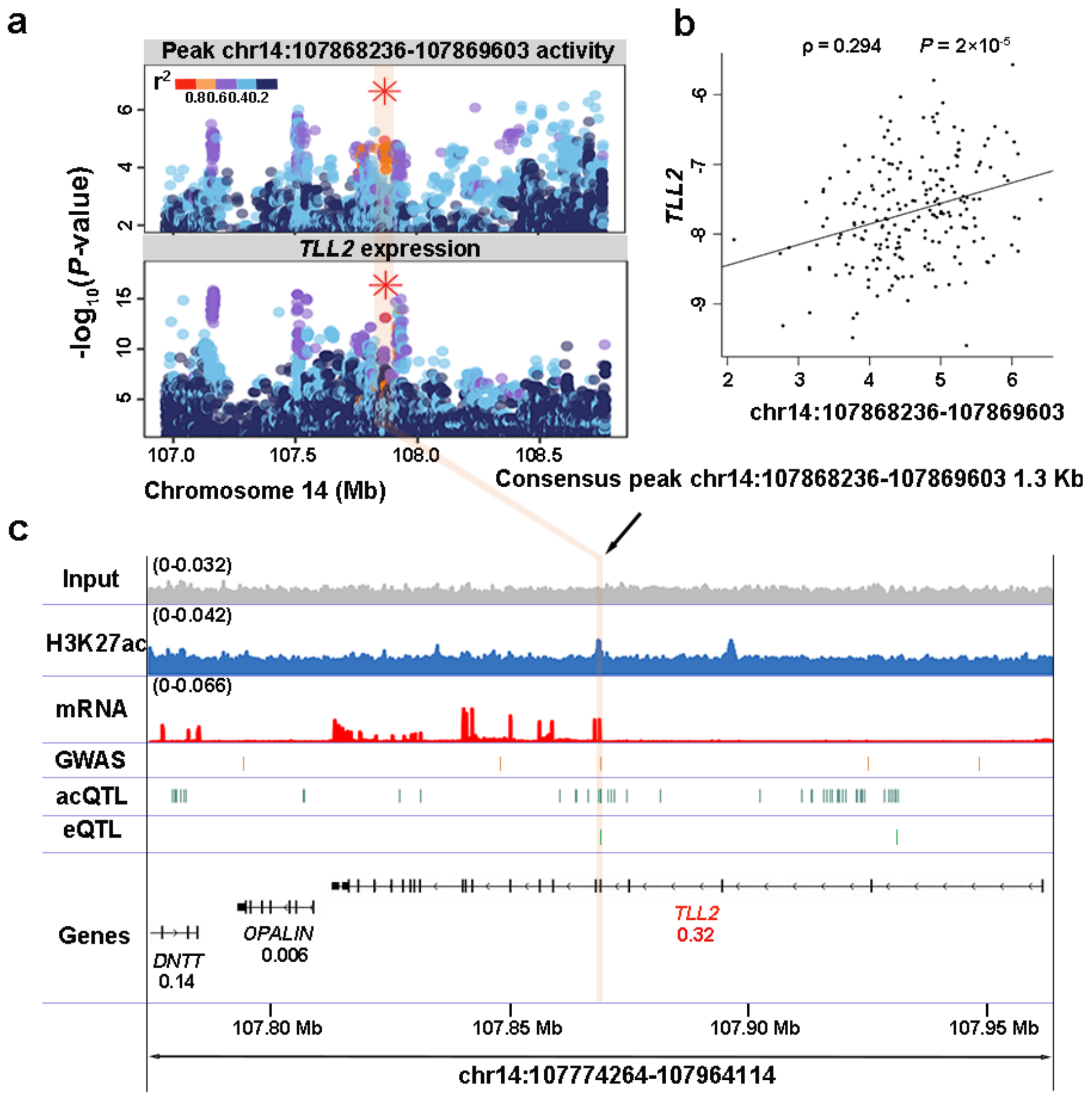

a Association plot for the peak activity (chr14:107868236-107869603) and gene expression (*TLL2*). The x-axis of the association plot represents the genomic range and the y-axis represents the $-\log_{10}(P\text{-value})$ for the association analysis. The candidate functional variant 14_107869191 is denoted by an asterisk and the peak is highlighted by a transparent orange rectangle. Dots represent all variants within the genomic range. The pairwise linkage disequilibrium (r^2) between the lead variant for the corresponding molecular phenotype and all genomic variants is indicated by distinct colors. **b** The correlation plot between the gene expression of *TLL2* and the peak activity of chr14:107868236-107869603. The x-axis and the y-axis represent the normalized signals for the peak and the gene, respectively. The solid black line represents the fitted curve, and the corresponding correlation coefficient and *P*-value are denoted (Spearman test). **c** Genome browser views of the comprehensive molecular profile of the region harboring candidate functional variants for hematocrit and red blood cell count. The x-axis displays the genomic range, and the y-axis contains several tracks of

data. The GWAS track displays the published variant from GWAS analysis, and the acQTL track shows candidate variants within 95% confidence interval for the consensus peak chr14:107868236-107869603 based on fine-mapping analysis. The consensus peak is highlighted by a transparent orange rectangle. The eQTL track depicts candidate variants within 95% confidence interval for the *TLL2* gene. The TPM values for each gene are displayed in gene track, with *TLL2* gene highlighted in red. The genomic annotations utilized were sourced from the Ensembl database (version 1.98 of the pig GTF file).

Supplementary Fig. 9: Comparison of H3K27ac signals across tissues.

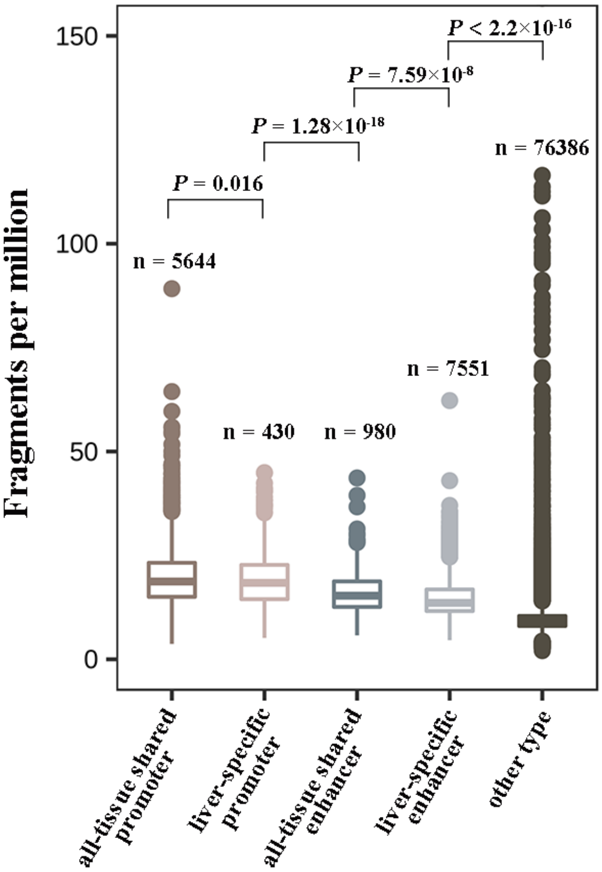

The distribution of H3K27ac signals of peaks group by chromatin states. The x-axis represents different groups. The y-axis represents peak activity measured by the average fragments per million. The significance of the difference between two group was calculated by t-test, and the resulting *P*-values are presented. The boxplots display the median, the 25th and 75th percentiles. The whiskers indicate the minimum and maximum values, and outliers are shown as points outside the ends of the whiskers.
