# Supplementary material for: Omics-based construction of regulatory variants can be applied to help decipher pig liver-related traits
Source: Commun Biol. 2024 Mar 29;7:381. doi: 10.1038/s42003-024-06050-7 (PMC10980749; doi:10.1038/s42003-024-06050-7)
Supplement: Supplementary file 3 — Description of additional supplementary files [file 42003_2024_6050_MOESM3_ESM.docx]

Description of Additional Supplementary Files

**File name:** Supplementary Data 1

**Description:** : H3K27ac ChIP-seq quality control.

**File name:** Supplementary Data 2

**Description:** The position of consensus superenhancers.

**File name:** Supplementary Data 3

**Description:** Predicted polyadenylated enhancer RNAs (eRNAs).

**File name:** Supplementary Data 4

**Description:** Cis-acQTLs.

**File name:** Supplementary Data 5

**Description:** Trans-acQTLs.

**File name:** Supplementary Data 6

**Description:** Allelic imbalance analysis of lead variants for acQTLs.

**File name:** Supplementary Data 7

**Description:** Lead variants for acQTLs were predicted to LOSE or GAIN the binding of TFs.

**File name:** Supplementary Data 8

**Description:** Lead variants for acQTLs were predicted to WEAKEN or ENHANCE the binding of TFs.

**File name:** Supplementary Data 9

**Description:** Physical contact from Hi-C.

**File name:** Supplementary Data 10

**Description:** Cis-eQTLs.

**File name:** Supplementary Data 11

**Description:** Trans-eQTLs.

**File name:** Supplementary Data 12

**Description:** The position of eQTLs relative to their target genes.

**File name:** Supplementary Data 13

**Description:** Colocalization of eQTLs and acQTLs.

**File name:** Supplementary Data 14

**Description:** Identification of putative functional genes and regulatory elements for GWAS variants of pig complex traits from the ISwine database.

**File name:** Supplementary Data 15

**Description:** Summary table for Supplementary Data 14.

**File name:** Supplementary Data 16

**Description:** H3K27ac peak coordinates.
