# Supplementary material for: Omics-based construction of regulatory variants can be applied to help decipher pig liver-related traits
Source: Commun Biol. 2024 Mar 29;7:381. doi: 10.1038/s42003-024-06050-7 (PMC10980749; doi:10.1038/s42003-024-06050-7)
Supplement: Supplementary file 5 — Reporting summary [file 42003_2024_6050_MOESM5_ESM.pdf]

nature portfolio

Corresponding author(s): Lusheng Huang, Bin Yang

Last updated by author(s): Feb 22, 2024

Reporting Summary

Nature Portfolio wishes to improve the reproducibility of the work that we publish. This form provides structure for consistency and transparency in reporting. For further information on Nature Portfolio policies, see our Editorial Policies and the Editorial Policy Checklist.

Statistics

For all statistical analyses, confirm that the following items are present in the figure legend, table legend, main text, or Methods section.

n/a	Confirmed	
☐	☒	The exact sample size (*n*) for each experimental group/condition, given as a discrete number and unit of measurement
☐	☒	A statement on whether measurements were taken from distinct samples or whether the same sample was measured repeatedly
☐	☒	The statistical test(s) used AND whether they are one- or two-sided<br>*Only common tests should be described solely by name; describe more complex techniques in the Methods section.*
☐	☒	A description of all covariates tested
☐	☒	A description of any assumptions or corrections, such as tests of normality and adjustment for multiple comparisons
☐	☒	A full description of the statistical parameters including central tendency (e.g. means) or other basic estimates (e.g. regression coefficient) AND variation (e.g. standard deviation) or associated estimates of uncertainty (e.g. confidence intervals)
☐	☒	For null hypothesis testing, the test statistic (e.g. *F*, *t*, *r*) with confidence intervals, effect sizes, degrees of freedom and *P* value noted<br>*Give P values as exact values whenever suitable.*
☐	☒	For Bayesian analysis, information on the choice of priors and Markov chain Monte Carlo settings
☐	☒	For hierarchical and complex designs, identification of the appropriate level for tests and full reporting of outcomes
☐	☒	Estimates of effect sizes (e.g. Cohen's *d*, Pearson's *r*), indicating how they were calculated

Our web collection on statistics for biologists contains articles on many of the points above.

Software and code

Policy information about availability of computer code

Data collection	No software was used for data collection
Data analysis	BWA v0.7.17,STAR v2.7.1a,Samtools v1.9,Platypus v0.8.1, PLINK v1.9,Beagle v0.40, Stringtie v1.3.6, FeatureCounts v1.5.3, Sambamba v0.8.1, Picard v1.119, MACS2 v2.1.1,DiffBind v2.10.0, Bedtools v2.27.0, verifyBamID v2.0.1, RSeQC v2.6.4, ChIPseeker v1.12.1, ROSE v1.3.1, edgeR v2.2.6, GCTA v1.9.0, PEER v1.3, Limma v3.38.3, FastQTL v6p, QTLtools v1.3.1, CAVIAR v2.2, WASP v0.3.4, GATK v.4.2, FIMO v4.11.2, LIMIX v2.0.4, GEMMA v.0.97, Coloc v5.

For manuscripts utilizing custom algorithms or software that are central to the research but not yet described in published literature, software must be made available to editors and reviewers. We strongly encourage code deposition in a community repository (e.g. GitHub). See the Nature Portfolio guidelines for submitting code & software for further information.

Data

Policy information about availability of data

All manuscripts must include a data availability statement. This statement should provide the following information, where applicable:

- Accession codes, unique identifiers, or web links for publicly available datasets
- A description of any restrictions on data availability
- For clinical datasets or third party data, please ensure that the statement adheres to our policy

All RNA-seq data and ChIP-seq data have been deposited in the China National Center for Bioinformation (CNCB, https://www.cncb.ac.cn/) under project number PRJCA022379. The accession numbers of the GSA database under PRJCA022379 are CRA014924, CRA014923 and CRA014930.

Research involving human participants, their data, or biological material

Policy information about studies with human participants or human data. See also policy information about sex, gender (identity/presentation), and sexual orientation and race, ethnicity and racism.

Reporting on sex and gender	NA
Reporting on race, ethnicity, or other socially relevant groupings	NA
Population characteristics	NA
Recruitment	NA
Ethics oversight	NA

Note that full information on the approval of the study protocol must also be provided in the manuscript.

Field-specific reporting

Please select the one below that is the best fit for your research. If you are not sure, read the appropriate sections before making your selection.

☒ Life sciences ☐ Behavioural & social sciences ☐ Ecological, evolutionary & environmental sciences

For a reference copy of the document with all sections, see nature.com/documents/nr-reporting-summary-flat.pdf

Life sciences study design

All studies must disclose on these points even when the disclosure is negative.

Sample size	256 for RNA-seq, 292 for H3K27ac ChIP-seq, 321 for GWAS analysis.
Data exclusions	Samples that were predicted to be swapped or contaminated will be removed according to the result of verifyBamID.
Replication	Peaks identified by H3K27ac ChIP-seq need to be replicated in at least three samples.
Randomization	NA
Blinding	NA

Reporting for specific materials, systems and methods

We require information from authors about some types of materials, experimental systems and methods used in many studies. Here, indicate whether each material, system or method listed is relevant to your study. If you are not sure if a list item applies to your research, read the appropriate section before selecting a response.

Materials & experimental systems

n/a	Involved in the study
☐	☒ Antibodies
☒	☐ Eukaryotic cell lines
☒	☐ Palaeontology and archaeology
☐	☒ Animals and other organisms
☒	☐ Clinical data
☒	☐ Dual use research of concern
☒	☐ Plants

Methods

n/a	Involved in the study
☐	☒ ChIP-seq
☒	☐ Flow cytometry
☒	☐ MRI-based neuroimaging

Antibodies

Antibodies used	H3K27ac antibody (active motif, 39133)
Validation	According to the statement from manufacture's website, H3K27ac antibody was test by ChIP using 30 µg of HAP1 myeloid leukemia cell chromatin. ChIP DNA was sequenced and sequence tags were mapped to identify Histone H3K27ac binding sites.

Animals and other research organisms

Policy information about studies involving animals; ARRIVE guidelines recommended for reporting animal research, and Sex and Gender in Research

Laboratory animals	The sixth (F6) generation pigs were from a heterogeneous population generated by crossing eight founder breeds including four aboriginal Chinese breeds (Erhualian, Laiwu, Bama Xiang, and Tibetan) and four highly selected international commercial breeds (Duroc, Large White, Landrace, and Pietrain). Liver samples from 292 individuals were collected.
Wild animals	NA
Reporting on sex	NA
Field-collected samples	NA
Ethics oversight	All procedures involving animals followed the guidelines for the care and use of experimental animals established by the Ministry of Agriculture of China. The ethics committee of Jiangxi Agricultural University specifically approved this study.

Note that full information on the approval of the study protocol must also be provided in the manuscript.

Plants

Seed stocks	NA
Novel plant genotypes	NA
Authentication	NA

ChIP-seq

Data deposition

☒ Confirm that both raw and final processed data have been deposited in a public database such as GEO.

☒ Confirm that you have deposited or provided access to graph files (e.g. BED files) for the called peaks.

Data access links<br>*May remain private before publication.*	All sequencing data have been deposited in the China National Center for Bioinformation (CNCB, https://www.cncb.ac.cn/) under project number PRJCA022379.
Files in database submission	ChIP sequencing data; RNA sequencing data; Peak position.

Genome browser session (e.g. UCSC)	NA

Methodology

Replicates	292 H3K27ac ChIP-seq data and 256 RNA-seq data from pig liver
Sequencing depth	A 150-bp paired-end strategy was employed fro RNA-seq; A 50-bp single end strategy was employed fro ChIP-seq. An average of 27.4 million uniquely mapped reads per sample for H3K27ac. An average of 40 million uniquely mapped reads per sample for RNA.
Antibodies	H3K27ac antibody (active motif, 39133)
Peak calling parameters	MACS callpeak -t ${Sample}_ac_final_sort.bam -c ${Sample}_input_final_sort.bam -n ${Sample}_ac -g 2.50e9 -p 1e-2 -f BAM --nomodel --extsize ${name}_temp --keep-dup all -B --SPMR
Data quality	The reads coverage was calculated using Bedtools, and peaks were retained if the log2 reads per million (log2RPM) was > 0 in at least 3 samples, yielding 91,011 raw peaks. To ensure consistency between acQTL and eQTL mapping analyses, FPM (fragments per million, similar to transcript per million from RNA) was used to represent the activity of H3K27ac peaks, and 90,991 consensus peaks satisfied the further filtering criteria from the GTEx project.
Software	MACS2 v2.1.1,DiffBind v2.10.0, Bedtools v2.27.0, ChIPseeker v1.12.1
